# Dosage Comparison of Snake Anti-Venomon Coagulopathy 

**Published:** 2014

**Authors:** Ali Hassan Rahmani, Amir Jalali, Mohammad Hassan Alemzadeh –Ansari, Mina Tafazoli, Fakher Rahim

**Affiliations:** a *Department of Clinical Toxicology, Razi Hospital, Jundishapur University of Medical Sciences,Ahvaz, Iran. *; b *Department of Pharmacology and Toxicology, School of Pharmacy, Jundishapur University of Medical Sciences, Ahvaz, Iran. *; c *Toxicology Research Center, Jundishapur University of Medical Sciences, Ahvaz, Iran. *

**Keywords:** Snake bite, Anti-snake venom, Iranian snakes, Coagulopathy complication

## Abstract

This study was done to determine whether high or low dose ofanti-snake venom (ASV) is better incoagulopathy invictims of envenoming by vipers. This retrospective study was conducted on the 154 patients (Mean age ± SD, Range) of viper snake bites who were referred to the emergency ward of Razi Hospital, Ahvaz, Iran over 2 years period (2004-2006).According to the treatment dosage the patients were divided in two groups include group 1(78 cases), low dose regimen and group 2 (76 cases), high dose one. In group 1, the treatment was performed by administration of 4 to 6 vials of ASV through intravenous infusion.In group 2, the patients were given 5 to 10 vials of ASV as an initial dose. In low dose regimen, the number of received packed red blood cell was higher (14 vs. 3) in comparison with high dose group. The number of ASV vials the patients received was 5.5and 21.06 in group 1 and 2, respectively (5.5±1.7 vs. 21.06±10.89; p < 0.01).The difference in frequency of coagulopathy complications, and need for using packed red blood cell were statistically significant(96.2% and 17.9% in group 1 vs. 34.2% and 3.9% in group 2, p < 0.01).It seems that cautious usage of high dose of ASV (10-20 vials) without very special concerns about the cost, dose, and without hazardous side effects is essential for the routine management of sever snake envenoming.

## Introduction

At least 421,000 envenoming and 20,000 deaths occur worldwideeach year due to snake bite (1).The highest rates of mortality and morbidity are observed in Asia among developing countries (2). 

ASV is most effective when used in appropriate dosage and appropriate indication (3). However, there is conflicting advice about the appropriate dosing and the frequency of its administration and duration of therapy among physicians. Recommending a universal initial dose required to neutralize the circulating venom is difficult, because there is no well-known end point available in anti-venom administration against snake bit envenoming (4-6). The recommended initial doses are ranging from one to four anti-venoms in Australia (7). Even when venom is neutralized by anti-venom, the coagulopathy state is recovered after a delay. 

The available anti-venom is produced against the most dangerous Iranian snakes Echis carinatus, Naja naja oxiana, Vipera lebtina, Vipera albicornuta, Agkistrodn halys, Pesudocerates persicus to Razi institute instruction.This anti-venin is currently considered asthe standard treatment protocol for the treatment of snake envenoming in Iran. The available polyvalent ASV is effective against hematotoxic and neurotoxic signs of Iranian snakes. This ASV is expensive, scarce, and it may be associated with allergic reactions to serum. The efficacy of this anti-venom has not previously been determined and there is notcomplete agreement about its effectiveness among Iranian physicians. Many physicians consider giving too small doses because of a fear of allergic reactions.

Venomous snake bites are common and deadly emergencies during the warm months in the southwestern of Iran, Khuzestan with the warm and hot temperature conditions. Venomous snakes in this area mainly have hemotoxic and/or neurotoxic venom.

The Center of Toxic Emergency of General Hospital has the largest caseload of patients with severe envenoming by stings in south and southwest of Iran. According to presented records, snakebite is said to have accounted for about 30% of allpatients of the venomous animals’stings requiringa stay at this hospital. This record reaches mostly 300 cases manually. Therefore, severe local snake envenoming is a common presentation to Razi Hospital. Patients with venomous snake bitesadmitted to the hospital present mainly signs and symptoms ranging from bleeding, respiratory depression, sweating to life-threatening coagulophathy, renal failure, and shock. 

Coagulopathy is a common manifestation of severe snake envenoming (8-9).Therefore, the aim of this study was to evaluate the effectiveness of two different dosage protocols of anti-venom administration on the outcomes of patients with severe local snake envenoming especially the coagulopathy state.

## Experimental

*Study design and population*

This retrospective study was conducted onthe 154 patients (Mean age ± SD, Range) of viper snake bites who were referred tothe emergencyward of Razi Hospital, Ahvaz, Iran over 2 years period (2004-2006). All patients were identified with a diagnosis of coagulopathy following severe viper envenoming and included in this study.This research was approved by the Jundishapur University of medical sciences Ethical Committee, Ahvaz, Iran.

*Materials*

The anti-venom was a sterile preparation contains equine immunoglobulin fragments F(ab)2. This lyophilized10mL polyvalent is an enzyme refined serum that produced against the most dangerous Iranian snakes including *Echis carinatus, Naja naja oxiana , Vipera lebtina, Vipera albicornuta, Agkistrodn halys and Pesudocerates *(Razi Vaccine and Serum Research Institute, Karaj, Iran).

*Methods*

Snake envenoming was defined by patientreport, correlation between clinical manifestations and recognition of snakes by patients and bystanders. There is no available venom-specific enzyme immunoassay, so snake species have not been identified in all cases. The majority of patients were stung by *Echis carinatus *(almost 90% of cases).For the prevention of hypersensitivity reactions to polyvalent ASV, corticosteroids, H1 and H2 anti-histamine receptor blockers, and epinephrine were available at the patient›s bedside (10). Detail of demographic information (age and sex), residency (rural or urban), findings of physical examination including site of bite, local reaction at the bite site and systemic features, and records of investigations including arterial blood gases, serum biochemistry, complete blood count, and coagulation profile were noted, Details of the dosage and schedules of SAV were and related information (number of used vials, interval time between bite and initiation of ASV administration, hypersensitivity reactions to ASV, coagulopathy complications, administration of FFP and packed red blood cell, and hospitalization time) also were reviewed. The coagulopathy was defined by the presence of platelet count <150,000 mm3, prothrombin time above normal, or fibrinogen level <150 mg/dL conditions (11).

According to the treatment dosage the patients were divided in two groups include group 1, low dose regimen and group 2, high dose one. In group 1, the treatment was performed by administration of 4 to 6 vials of ASV through intravenous infusion (12). Then, prothrombin time, fibrinogen level, and platelet counts were determined. Then, the patient was evaluated for local injury. These parameters, in addition to clinical evaluation, were used for the purpose of screening for coagulopathy. For achieving endpoint, three maintenance doses of two vials every six hours were given. The endpoint state was defined as arrest of local tissue manifestations and return of prothrombin time, fibrinogen level, platelet counts, and systemic signs to normal. If the endpoint state was achieved, the patient could be discharged and after three days he or she should be visited in follow up clinic; and if the endpoint was not achieved, the patient would be received additional doses of ASV. 

In group 2, the patients were given 5 to 10 vials of ASV as an initial dose.Following the initial dose,prothrombin time, fibrinogen level, and platelet counts were measured. Then, the patients were evaluated for local injury. All hematological and clinical monitoring were same as grooup1. Against prior method, the patients should be received additional doses ofASV if coagulopathy, thrombocytopenia, and worsening tissue injury were persisted.The number of used vials reached even 40. The evaluation daysreached even6.Hypersensitivity reactions to ASV were defined solely as urticaria in most cases.In group 1, administration was performed without paying much attention to clinical manifestations, whereas in second group, it was mainly based on clinical manifestations and coagulopathy.Snakes were identified either by direct examination ofthe snakes when brought by the patients or on the basis ofthe signs, symptoms, and results of the investigations. 

*Statistical analysis*

The data wasanalyzed using SPSS 11.5 (SPSS Inc., Chicago, IL, USA). The data were expressed as mean±standard deviation (SD) ornumber and percentage were suitable. The Chi square and the Student’s t-tests to find the significant difference when were appropriate. p< 0.05 is considered statistically significant.

## Results

Most of the patients were <30 years age. Of 154 patients identified with sever snake bites, 117 (76%) were male, and 37 (24%) were female. In the study group of 154 patients, 111 (72.1%) had been bitten on the lower limb, 41 (26.6%) had been bitten on the upper limb and the rest two cases (1.3%) had been bitten on the trunk. Most bites happened in rural areas 99(64.3%) (Table 1).

**Table 1:** Baseline characteristics of 154 cases of envenoming patients

**Variables**		**p-value**	**Total (n=154)**	
Gender	Male	0.02**	117 (76%)	
	Female		37 (24%)
Residence	Rural	0.61	99 (64.3%)	
	Urban		55 (35.7%)
Time of bite	Day	0.50	56 (36.4%)	
	Night		98 (63.6%)
Site of bite	Upper limb	0.04▲	41 (26.6%)	
	Trunk		2 (1.3%)
	Lower limb		111 (72.1%)

** p<0.05 was considered significant

It took less than 24 hours from the time of bite to administration of the first dose of anti-venom in 68 (85.9%) and 75 cases (98.7%) in group 1 and 2, respectively. All patients were successfully treated and recovered completely. 

Of 78 cases who sustainedsevere envenoming in low dose regimen, the number of received packed red blood cell was higher (14 vs. 3) in comparison with high dose group. The number of received clotting factor (FFP and/or cryoprecipitate) units was 24 in group 1, but it was 14 in group 2. The number of ASV vials the patients received was 5.5±1.7 and 21.06±10.89 in group 1 and 2, respectively (5.5 ± 1.7 vs. 21.06 ± 10.89; p < 0.01). No significant difference was seen in number of used FFP units, hypersensitivity to ASV, and time of hospital stay between two methods. The difference in frequency of coagulopathy complications, and need for using packed red blood cell were statistically significant between two groups (96.2% and 17.9% in group 1 vs. 3.9% in group 2, p < 0.01).Coagulopathy as an important factor of snake envenoming was decreased significantly (p < 0.01) in group 2. The coagulopathy state was remained in 75 (96.2%) and 26 (34.2%) ofcases in group 1 and 2, respectively (p < 0.01) (Table 2).

**Table 2:** Data related to management of patients

**Parameters**	**Low dose; N (%)**	**High dose; N (%)**	**p-value**
ASV*, Mean ± SD	5.5±1.7	21.06±10.89	<0.01**
Number of FFP, No. (%)	24 (30.8%)	14 (18.4%)	0.09
Number of packed red blood cell, No. (%)	14 (17.9%)	3 (3.9%)	<0.01**
Hypersensivity reaction to ASV, No. (%)	8 (10.3%)	4(5.3%)	0.37
Coagulopathy state, No. (%)	75 (96.2%)	26 (34.2%)	<0.01**
Hospital stay in days*, No. (%)	3.5±0.91	3.5±0.9	0.99

The number of used Anti-snake venom (ASV) and hospital stay in days were presented as ± standard deviation; ** P<0.05 was considered significant; FFP, Fresh Frozen Plasma

## Discussion

Hemotoxic snake envenoming, mainly coagulopathy is one of the most important causes of snake bite fatality in Iran, especially in Khuzestan and is mainly due to the Viperidae family, which include *Echis carinatus *and *Agkistrodon halys *commonly referred to as the Viper.This study was done to evaluate the efficacy and complications of two methods of ASV administration: administration of additional doses of available ASV, together with controlling manifestations of patients with severe snake envenoming clinically and experimentally versus administration of additional doses of ASV when complications continued or occurred. The results showed that administration of 5-10 vials of ASV as an initial dose is effective to decrease incidence rate of coagulopathy in patients with severe snakes envenoming. 

The effect of venom fractions from viper family on blood coagulation and ADP-induced platelet aggregation were studied previously, and showed that the fractions have coagulant activity in PT test and procoagulant activity as well (13).

The venom of vipers causes life-threatening signs such as micro-angiopathichaemolysis, coagulopathy and acute renal failure (14). Restoration of coagulability and platelet function can be accelerated by giving FFP,fibrinogen, fresh whole blood or platelet concentrates(3). The measured parameters including number of FFP units, number of packed red blood cell, coagulopathy state, and hypersensitivity reaction to ASV are used routinely to evaluate anti-venin treatment in Viperidae cases. These results had profound implications for high dose anti-venom therapy of severe snake envenoming, at least envenoming resulting from local viper snake bites. This result is supported by previous *in-vitro *(15), animal (16), and clinical studies (17, 18).

The significance decrease of packed red blood cell in group 2 following high dose of ASV, indicated that this treatment is not essential for severe viper snake envenoming (19, 20).This controlled clinical study showed that administration of FFP containing clotting factors should be necessary if a neutralizing dose of anti-venom is administered. This is consistent with the available recommendation (21). Furthermore; the results recommended that larger doses of anti-venom be required to prevent afibrino genemia with serious consequences, for prolonged periods. This conclusion isn’t recommended in active hemorrhagic condition or unusual hepatic repletion of clotting factors. Tariang and colleagues reported that the neurotoxic envenomation requires higher dose of ASV (22), while Paul and colleagues reported higher mortality rate and higher percentage of cases requiring dialysis in higher doses (23).This difference may be due to neurotoxic snakes in Tariang᾽s study and haemotoxic snakes in Paul᾽s study. Furthermore, the effects of two different dosage protocols on the outcomes of patients with severe neurotoxic snake envenoming were studied in a short report. This report suggested that low dose of snake anti-venom be as effective as high dose in patients with severe neurotoxic snake envenoming in India (24). In this country, snake bites occur predominantly by Elapid snakes (cobras) with neurotoxicity manifestations.In conclusion, there is an urgent condition to receive high dose of anti-venom in the regions like Khuzestan where Viperidae envenoming mainly *Echis carinatus *is a major public health issue.

In the present study, 76% of the patients were male as compared to 24% of females. In other researches, this male preponderance was seen. Hansdak *et al. *had found that snake bites were 2.5 times more common in males (25). Meyer *et al. *found that 85% of patients were male (26). In the study done by Narvencar, 90% of patients were male (27). This ratio may be probably due tothe fact that menhave occupational exposures andtheyspend more time outside the home.

Although snake bite during the night is more than during the day, no significant difference was seen. Al-Lawatia and colleagues reported that most of the bites happened in the afternoon or evening (28), whereas Frangides and colleagues reported that the largest incidence of bite was during the warmest midday hours (29).

The present study revealed that most snake bites occurred in rural areas, which other studies have also showed this fact (30, 31). The high incidence of snake bites in rural areas is probably due to their lifestyle and occupational exposure as farmers or herdsman. In our study, site of bite was more significantly lower limbs. In the study carried out in Khuzestan previously, 71.8% of snake bites wereonfoot (32). This result is supported by previous studies (22, 33). ASV, particularly when given intravenously, not infrequently results in early reactions, ranges from rash and urticaria to fatal anaphylaxis. Pyrogenic and late serum sickness-type reactions can also cause distressing symptoms (5-6). The extra anti-venoms were reported to be more allergenic, with acute reactions seen in more than 20% of patients (10, 34). As showed, administration of available ASV was safe since hepersensitivity reaction was only seen in 8 (10.3%) cases in 2004 and 4 cases (5.3%) in 2006, following administration of high-dose vials. Theseresults may also suggest that the incidence of serum sickness be unrelated to dose. This finding is contrary to previous studies and case reports of severe reactions to antivenin suggesting that dosing regimens cause a fear of polyvalent anti-venin therapy and the reluctance of clinicians to administer high doses of anti-venin (10, 35-37). There are four limitations that need to be highlighted and addressed: 1) the clinical effects of bites are not carefully studied. 2) The viper variation in venom components, yield and severity leads to different clinical presentations include coagulopathy sign. 3) Pre-existing haematological or platelet disorders. 

## Conclusion

The effectiveness of high dose of snake anti-venom was showed in coagulopathy in patients with severe snakes envenoming. However, the titer and efficacy of available anti-venom shouldbe evaluated experimentally. Although some authors demonstrated that lower doses are as effective as high doses which are cheaper and safer, it seems that cautious usage of high dose of ASV (10-20 vials) without very special concerns about the cost, dose, and without hazardous side effects is essential for the routine management of sever snake envenoming. 

## References

[1] Kasturiratne A, Wickremashinghe R, de Silva N, Gunawardena NK, Pathmeswaran A, Premaratna R, Savioli L, Lalloo DG, de Silva HG (2008). The global burden of snakebite: a literature analysis and modelling based on regional estimates of envenoming and deaths. PLoS Med..

[2] Harrison RA, Harrison RA, Hargreaves A, Wagstaff SC, Faragher BandLalloo DG (2009). Snake envenoming: a disease of poverty. PLoS Negl. Trop. Dis..

[3] (1999). WHO/SEARO Guidelines for the clinical management of snake bites in the Southeast Asian region. Southeast Asian J. Trop. Med. Pub. Health.

[4] Buntain WL (1983). Successful venomous snakebite neutralization with massive anti-venin infusion in a child. J Trauma..

[5] Hanvivatvong O, Mahasandana S, Karnchanachetanee C (1997). Kinetic study of russell›s viper venom in envenomed patients. Am J. Trop. Med. Hyg..

[6] Ariaratnam CA, Sheriff MHR, Arambepola C, Theakston RDG, Warrell DA (2009). Syndromic approach to treatment of snake bite in Sri Lanka based on results of a prospective national hospital-based survey of patients envenomed by identified snakes. Am J. Trop. Med. Hyg..

[7] Yeung JM, Daly FFS, Little M, Murray LM, Jelinek GA (2004). Anti-venom dosing in 35 patients with severe brown snake (Pseudonaja) envenoming in Western Australia over 10 years. Med J. Aust..

[8] Milani JR, Jorge MT, de Campos FP, Martins FP, Bousso A, Cardoso JL, Ribeiro LA, Fan HW, França FO, Sano-Martins IS, Cardoso D, Ide Fernandez C, Fernandes JC, Aldred VL, Sandoval MP, Puorto G, Theakston RD, Warrell DA (1997). Snake bites by the jararacucu (Bothrops jararacussu): clinicopathological studies of 29 proven cases in Sao Paulo State, Brazil. QJM: An Int J. Medicine.

[9] Isbister GK, Brown SG, MacDonald E, White J, Currie BJ (2008). Current use of Australian snake anti-venoms and frequency of immediate-type hypersensitivity reactions and anaphylaxis. Med J. Aust..

[10] Jurkovich GJ, Luterman A, McCullar A, Ramenofsky ML, Curreri PW (1988). Complications of crotalidae anti-venin therapy. J Trauma.

[11] Bogdan GM, Dart RC, Falbo SC, McNally JandSpaite D (2000). Recurrent coagulopathy after anti-venom treatment of crotalid snakebite. South Med J.

[12] Gold BS, Dart RC, Barish RA (2002). Bites of venomous snakes. New Engl J. Med..

[13] Mehdizadeh Kashani T, Vatanpour H, Zolfagharian H, Hooshdar Tehrani H, HeydariMH, Kobarfard F (2012). Partial Fractionation of Venoms from Two Iranian Vipers, Echiscarinatus and Cerastes persicus Fieldi and Evaluation of Their AntiplateletActivity. Iran J Pharm. Res..

[14] Schneemann M, Cathomas R, Laidlaw ST, El-Nahas AM, Theakston RDG, Warrell DA (2004). Life-threatening envenoming by the Saharan horned viper (Cerastes cerastes) causing micro-angiopathic haemolysis, coagulopathy and acute renal failure: clinical cases and review. QJM: An Int J.Medicine.

[15] Sprivulis P, Jelinek GA, Marshall L (1996). Efficacy and potency of anti-venoms in neutralizing the procoagulant effects of Australian snake venoms in dog and human plasma. Anaesth Intensive Care.

[16] Tibballs J, Sutherland S (1991). The efficacy of anti-venom in prevention of cardiovascular depression and coagulopathy induced by brown snake (Pseudonaja) species venom. Anaesth Intensive Care..

[17] Jelinek GA, Hamilton T, Hirsch RL (1991). Admissions for suspected snake bite to the perth adult teaching hospitals, 1979 to 1988. Med J. Aust..

[18] Barrett R, Little M (2003). Five years of snake envenoming in far north Queensland. Emerg Med. (Fremantle)..

[19] Isbister GK, Duffull SB, Brown SG (2009). Failure of anti-venom to improve recovery in Australian snakebite coagulopathy. QJM: An Int J. Medicine.

[20] Ferguson LA, Morling A, Moraes C, Baker A (2002). Investigation of coagulopathy in three cases of tiger snake (Notechisater occidentalis) envenomation. Pathology.

[21] White J (2002). Management of brown snake envenoming. Crit Care Resusc..

[22] Tariang DD, Philip PT, Alexander G, Macaden S, Jeyaseelan L, Peter JV, Cherian AM (1999). Randomized controlled trial on the effective dose of anti-snake venom in cases of snake bite with systemic envenomation. J Assoc. Phys. India.

[23] Paul V, Pratibha S, Prahlad KA, Earali J, Francis S, Lewis F (2004). High-dose anti-snake venom versus low-dose anti-snake venom in the treatment of poisonous snake bites--a critical study. J Assoc. Phys. India.

[24] Aggarwal R, Aggarwal AN, Gupta D, Behera D, Jindal SK (2005). Low dose of snake anti-venom is as effective as high dose in patients with severe neurotoxic snake envenoming. Emerg Med. J..

[25] Hansdak SG, Lallar KS, Pokharel P, ShayangwaP, Karki P, Koirala S (1998). A clinico-epidemiological study of snake bite in Nepal. Trop Doct..

[26] Meyer WP, Habib AG, Onayade A, Yakubu A, Smith DC, Nasidi A, Daudu IJ, Warrell DA, Theakston RD (1997). First clinical experiences with a new ovine Fab Echis ocellatus snake bite anti-venom in Nigeria: randomized comparative trial with Institute Pasteur Serum (Ipser) Africa anti-venom. Am J. Trop. Med. Hyg..

[27] Narvencar K (2006). Correlation between timing of ASV administration and complications in snake bites. J Assoc. Phys. India.

[28] Al-Lawati A, Al-Abri SS, Lalloo DG (2009). Epidemiology and outcome of snake bite cases evaluated at a Tertiary Care Hospital in Oman. J Infect. Public Health.

[29] Frangides CY, Koulouras V, Kouni SN, Tzortzatos GV, Nikolaou A, Pneumaticos J, Pierrakeas C, Niarchos C, Kounis NG, Koutsojannis CM (2006). Snake venom poisoning in Greece. Experiences with 147 cases. Eur. J. Intern. Med.

[30] Hayat AS, Khan AH, Shaikh TZ, Ghouri RA, Shaikh N (2008). Study of snake bite cases at Liaquat University Hospital Hyderabad/Jamshoro. Ayub. Med. Coll. Abbottabad..

[31] Gutierrez JM, Williams D, Fan HW, Warrell D A (2010). Snakebite envenoming from a global perspective: Towards an integrated approach. Toxicon.

[32] Alavi SM, Alavi L (2008). Epidemiology of animal bites and stings in Khuzestan, Iran, 1997-2006. J Infect. Public Health.

[33] Bochner R, Struchiner CJ (2003). Snake bite epidemiology in the last 100 years in Brazil: a review. Cad Saude. Publica..

[34] Tanen D, Ruha A, Graeme K, Curry S (2001). Epidemiology and hospital course of rattlesnake envenomations cared for at a tertiary referral center in Central Arizona. Acad Emerg. Med..

[35] Barratt SM (1999). A life-threatening anaphylactoid reaction to polyvalent anti-venom despite pretreatment. Med J. Aust..

[36] Sutherland SK (1999). A life-threatening anaphylactoid reaction to polyvalent anti-venom despite pretreatment. Med J. Aust..

[37] Arunanthy S, Hertzberg SR (1998). A life-threatening anaphylactoid reaction to polyvalent snake anti-venom despite pretreatment. Med J. Aust..

